# Exome sequencing study revealed novel susceptibility loci in subarachnoid hemorrhage (SAH)

**DOI:** 10.1186/s13041-020-00620-6

**Published:** 2020-05-25

**Authors:** Xiwa Hao, Jiangxia Pang, Ruiming Li, Lin Lv, Guorong Liu, Yuechun Li, Guojuan Cheng, Jingfen Zhang

**Affiliations:** Department of Neurology, Baotou Central Hospital, Baotou, China

**Keywords:** Subarachnoid hemorrhage (SAH), Whole-exome sequencing (WES), Genome-wide association analysis (GWAS), Rare variations, Pedigree analysis

## Abstract

**Aim:**

To expand our current understanding of the genetic basis of subarachnoid hemorrhage (SAH), and reveal the susceptibility genes in SAH risk.

**Methods:**

We conducted whole-exome sequencing (WES) in a cohort of 196 individuals, including 94 SAH patients and 94 controls, as well as 8 samples that belong to two pedigrees. Systematically examination for rare variations (through direct genotyping) and common variations (through genotyping and imputation) for SAHs were performed in this study.

**Results:**

A total of 16,029 single-nucleotide polymorphisms (SNPs) and 108,999 short indels were detected in all samples, and among them, 30 SNPs distributed on 17 genes presented a strong association signal with SAH. Two novel pathogenic gene variants were identified as associated risk loci, including mutation in *TPO* and *PALD1.* The statistical analysis for rare, damaging variations in SAHs identified several susceptibility genes which were involved in degradation of the extracellular matrix and transcription factor signal pathways. And 25 putative pathogenic genes for SAH were also identified basic on functional interaction network analysis with the published SAH-associated genes. Additionally, pedigree analysis revealed autosomal dominant inheritance of pathogenic genes.

**Conclusion:**

Systematical analysis revealed a key role for rare variations in SAH risk and discovered SNPs in new complex loci. Our study expanded the list of candidate genes associated with SAH risk, and will facilitate the investigation of disease-related mechanisms and potential clinical therapies.

## Introduction

Subarachnoid hemorrhage (SAH), the rarest but most fatal type of stroke, has shown an annual incidence of 8–10/100,000 persons (2007), 30-day case fatality of 35–45% in western countries [17, 20]. In China, annual incidence (per 100,000 persons) of SAH was 6.2, which is slightly lower than in western countries [40]. The majority of patients with SAH usually suffered from ruptured intracranial aneurysm (IA). SAH risk was considered to be related to smoking, hypertension, and poor socioeconomic status [2, 14]. Moreover, studies based on molecular mechanisms have shown that genetic factors also play an important role in the formation, growth and rupture of IA [6, 11, 24, 32, 38]. Therefore, IA is identified as a complex disease that influenced by various genes and environmental factors. Conducting early detection and intervention by identifying risk factors may facilitate to avoid the formation and rupture of IA, and is crucial for the reduced incidence of SAH [12]. However, comprehensive knowledge of pathogenic and ruptured mechanisms of IA has not yet been defined.

Some studies have focused on the pathogenic mechanisms of IA in China, and several susceptibility genes have been identified. Due to the limitation of technology and small sample size, it is still necessary to further study the genetic factors of IA in China. Although genome-wide association analysis (GWAS) studies have found some novel gene loci related to IAs, they can only explain part of the genetic risk. Most of the GWAS studies have focused on both unruptured and ruptured IAs, thus, the gene loci highly related to ruptured IAs have not been completely detected. Moreover, rare, damaging variants also play an important role in complex diseases. With advances in sequencing technology, genetic analysis is gradually extending to rare variants, which often have more obvious functional consequences of harmful phenotypes [13, 29].

In this study, we set out to systematically examine rare variation (through direct genotyping) and common variation (through genotyping and imputation) for SAHs by whole-exome sequencing (WES) in a cohort of 196 samples. Mendelian inheritance analysis for the SAH pedigrees was also performed to identified the susceptibility genes. Our study constitutes a detailed simultaneous assessment of causal variations in a large sample of SAHs, offer an opportunity to better understand both the biological and genetic architecture of this type of complex disease.

## Materials and methods

### Study cohorts

We prospectively collected 196 samples, including 8 samples that belong to two pedigrees from Central Hospital of Baotou. The cohorts included 94 SAH cases(with ruptured intracranial aneurysm confirmed by Digital Subtraction Angiography, DSA and computed tomography, CT) and 94 controls(for each case, 1 control without SAH will be sort for interview. Controls will be matched on the basis of the following criteria:

- gender (sex)
- 10-year age strata (ie 10–19, 20–29, etc)
- sector of suburb of residence in Baotou (North, East, etc).

Controls will be chosen from the *spouse, relative or friend* of patients without SAH who are currently in the same hospital as the case.). This study was approved by the Human Research Ethics Committee of Central Hospital of Baotou, and all participants provided written informed consent. Comprehensive clinical information was provided in Table S1, including height, weight, BMI, gender and age etc.

### Whole-exome sequencing

Genomic DNA was isolated from peripheral whole blood samples of participants by using Genomic DNA Extraction Kit (Invitrogen, South San Francisco, CA, USA). The Qubit 3.0 fluorometer and gel electrophoresis were used to evaluate DNA quantity and integrity, respectively. The sequencing paired-end libraries were constructed for each sample and captured using SureSelect Human All Exon V6 kit (Agilent Technologies, Santa Clara, CA, USA) following the manufacturer’s instructions. All libraries were sequenced on BGI-SEQ 500 platform at BGI to obtain a desired depth of ~100X. The sequencing depths of each sample are listed in Table S2.

### Whole-exome sequencing (WES) data processing and variant calling

To get high quality data, Trimmomatic [5] was used to filter out low-quality reads which contained adaptors, high base error rate (> 50%), and highly unknown base proportion (> 10%) from the raw sequencing data. The cleaned reads were aligned to human reference genome (UCSC hg19) by the Burrows-Wheeler Aligner-MEM (v.0.7.15) [26] with default parameters. All the aligned reads were further processed using Picard tools (v2.5.0) and Genome Analysis Toolkit (GATK, v3.7) [28] with default parameters, which included deduplication, base quality recalibration, and multiple-sequence realignment prior to mutation detection.

Variant calling was performed for all the samples by using the Haplotype Caller algorithm in GATK with the parameters “-stand_call_conf 30 -stand_emit_conf 10 -minPruning 3”. Each variant was filtered using GATK hard filters with the parameters “QD<2.0 || FS>60 || MQ<40 || MQRankSum<-12.5 || ReadPosRankSum<-8.0” for SNPs and “QD < 2.0 || FS > 200 || ReadPosRankSum < -20” for Indels to reduce the false positive rate. We then called genotypes jointly across all samples at the remaining sites, followed by genotype refinement using the BEAGLE imputation software (v5.0) [7]. The variants were subsequently annotated by multiple databases using the ANNOVAR tool [37].

### Sample quality control

The standard quality screening conducted independently in each sample included SNP and sample call rates (> 90%), Hardy–Weinberg equilibrium, Mendelian errors, gender inconsistencies and checks for population stratification. To obtain a high-quality set of samples, the outlier samples discovered using principal-component analysis in GCTA [39] were removed from further analysis.

### Association testing

The single marker association analyses with SAH were performed using an additive genetic model implemented in SNPTEST (http://www.stats.ox.ac.uk/~marchini/software/gwas/snptest.html) for the common SNPs (MAF > 10%). Age, sex, BMI, smoking, drinking, body fat, and diabetes were used as covariates in the analysis.

### Rare SNP filtering

We used different allele frequency threshold in several public population databases: 1000G (http://browser.1000genomes.org/index.html), ExAC, ESP etc., to filter out common variants. Then, only variants with frequency less than the thresholds in all these databases were considered as the rare SNPs of SAHs.

### Functional impact prediction

Each variant category has to be assessed with a specific set of tools to predict their functional impact. Here, we assumed that synonymous variants have no functional impact, and all the stop gain and stop loss variants were considered as the deleterious mutations. The functional predictions of missense variants were performed by seven computational methods (SIFT (Ng, 2001 #4097), Polyphen2 [1], MutationTaster [33], CADD [27], REVEL [21], M-CAP [22], LRT [10]). The pathogenicity of missense mutations was assumed if predicted pathogenic by at least five out of the computational methods. The dpsi score were employed to determine the pathogenicity of splicing mutations.

### Gene-based burden analysis

Gene-base test were performed for the rare, damaging variants. For each gene, we computed the burden of rare, damaging variants in SAH cases and controls, respectively. Fisher’s exact test was applied to determine the significantly associated genes in SAHs. Those genes with a *P*-value of less than 0.05 were identified as susceptibility genes in SAHs. SKAT-O [25] was also applied for burden test, which allowing for variants with opposite directions of effect to reside in the same gene.

### Inheritance analysis in pedigrees

The SNPs were called from the 2 pedigrees, and were further filtered as the filtering criterion of rare SNPs. Then, all the SNPs were subjected to functional impact prediction. Mendelian inheritance analysis was performed for the diseasing causing SNPs with 4 inheritance patterns, including (1) dominant inheritance pattern; (2) recessive inheritance pattern; (3) semi-dominant inheritance pattern; (4) compound heterozygote inheritance pattern.

### The network analysis

The SAH-associated genes were collected from the published studies. The STRING database and associated search tools [35] were used for identifying interacting partners of a list of SAH-associated genes. We employed the identified interacting partners as the candidate pathogenic genes in SAHs.

## Results

### Cohorts description and whole-exome sequencing

In this study, we performed ~100x whole-exome sequencing (WES) for 94 SAH cases, 94 controls, and 2 pedigrees. Comprehensive description of the height, weight, sex, age and the other clinical variables of the cohort are provided in Table S1. In brief, the SAH group included 55 hypertension, 10 diabetic, 11 hyperlipidemia, 46 smoker/former-smoker, and 22 drinker/former-drinker. The control group included 37 hypertension, 9 diabetic, 9 hyperlipidemia, 42 smoker/former-smoker, and 23 drinker/former-drinker. The statistics of the WES data was provided in Table S2 and S3, including effective bases, SNPs numbers, Indel numbers and Ti/Tv rate etc. for each sample.

We totally discovered 716,029 single-nucleotide polymorphisms (SNPs) and 108,999 short indels in all the samples. We then applied Genome Analysis Toolkit (GATK) VQSR for SNVs to distinguish true sites of genetic variation from sequencing artifacts. Then, 549,553 SNPs were remained, including 148,967 exonic SNPs, for the further analysis (See Method section, Table S4). Following sample quality control, the whole-exome sequences of 93 patients with SAH and 92 controls were jointly analyzed (See Method section).

### Imputation into GWAS

**Fig. 1:**
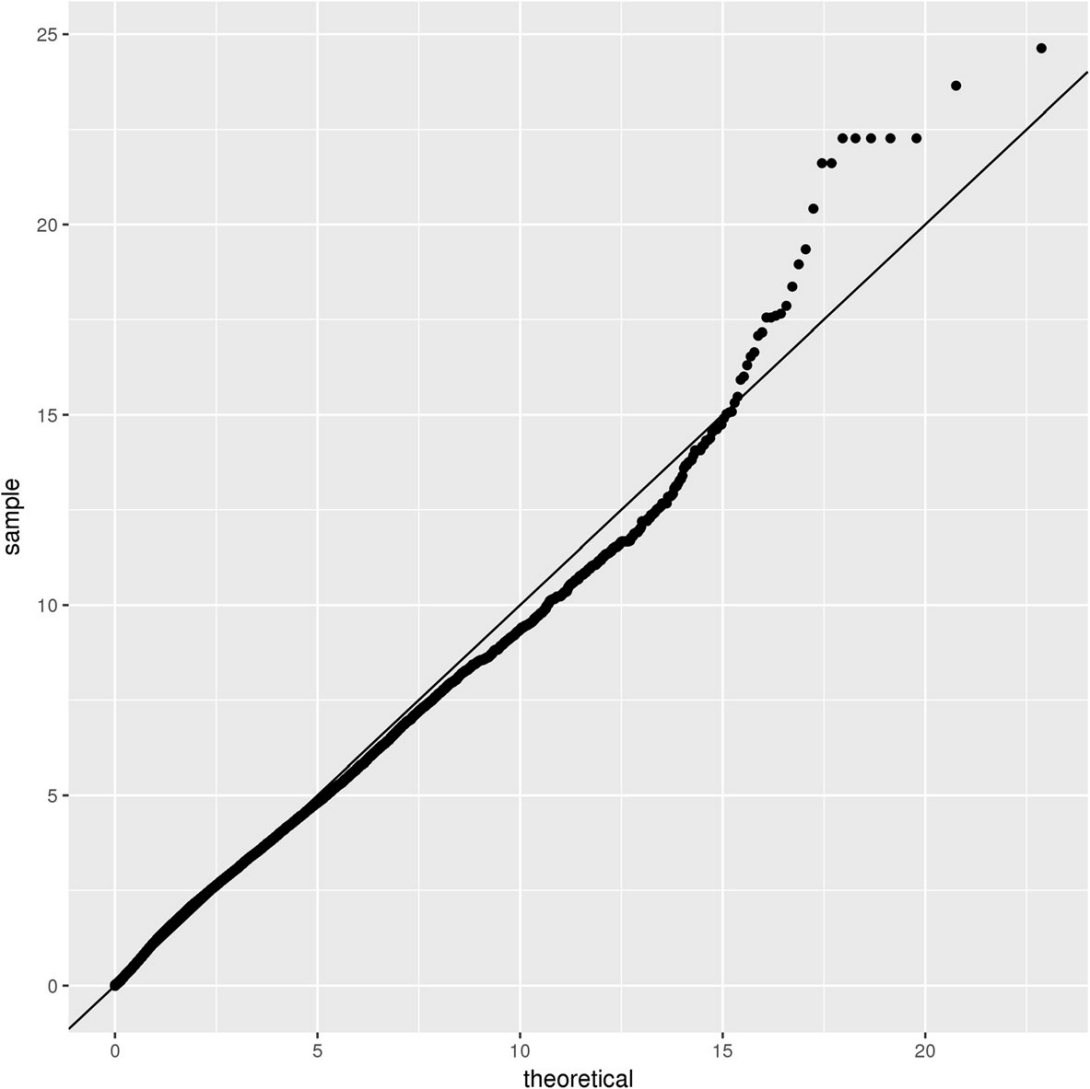
Quantile–quantile (Q–Q) plots of the meta-analyses of genome-wide association studies (GWAS) results for SAH

**Fig. 2:**
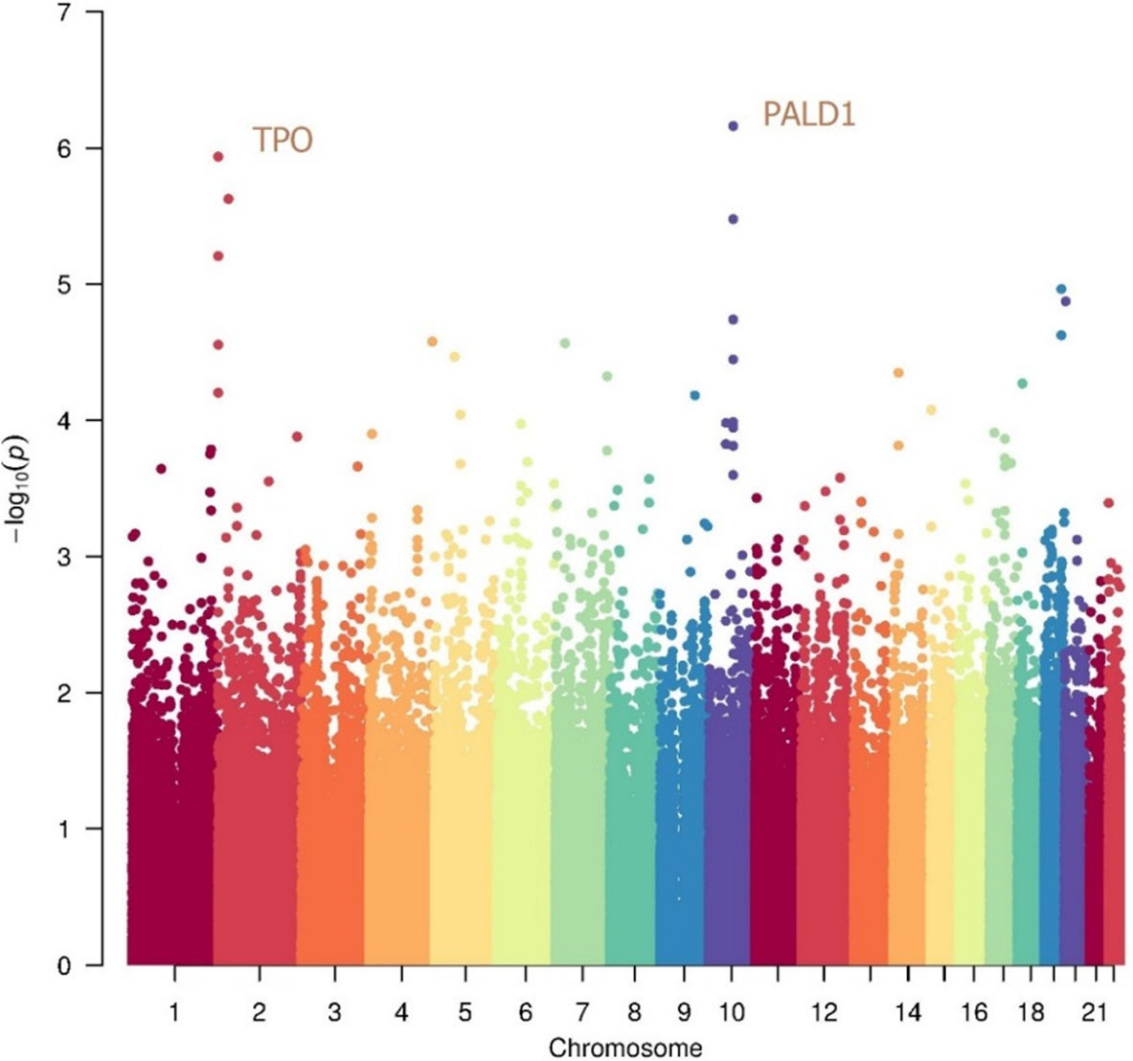
Manhattan plot depicting the GWAS results for SAH. Each dot represents a single-nucleotide polymorphism (SNP), with the chromosomal position on the x axis and the *P*-value on the y axis

For imputation purposes, we conducted a genome-wide single-variant analysis of the common SNPs (minor allele frequency, MAF > 0.1) comparing the 93 SAH cases and 92 controls. The associations with SAH risk were tested using logistic regression adjusted for sex, BMI, smoking, drinking, body fat, and diabetes as covariates. The genomic inflation factor (λ = 1.006) showed no evidence of inflated test statistics. There were 30 SNPs distributed on 17 genes presented a strong association signal with SAH (Table 1, Fig. 1). In these SNPs, three of them were in exon, and one in UTR3, and the other in intron. We obtained two loci reached genome-wide significance, within the introns of two genes *TPO* and *PALD1*, respectively (Fig. 2), implies a putative functional role in the pathogenesis of SAHs.

**Table 1:** SNPs with the strongest association with SAH from the GWAS results

Chr	Pos	Ref	Alt	Cases MAF	Controls MAF	OR	***P*** value	Function	Gene
10	72,300,743	G	C	0.087	0.274	3.97	6.94E-07	intronic	*PALD1*
2	1,437,410	C	T	0.245	0.484	2.90	1.16E-06	intronic	*TPO*
2	31,189,236	A	G	0.212	0.425	2.75	2.37E-06	intronic	*GALNT14*
2	31,189,304	C	T	0.212	0.425	2.75	2.37E-06	intronic	*GALNT14*
2	31,189,345	T	G	0.212	0.425	2.75	2.37E-06	intronic	*GALNT14*
2	31,189,401	G	A	0.212	0.425	2.75	2.37E-06	intronic	*GALNT14*
2	31,189,439	A	G	0.212	0.425	2.75	2.37E-06	intronic	*GALNT14*
10	72,306,967	T	C	0.174	0.366	2.74	3.33E-06	intronic	*PALD1*
10	72,306,978	A	C	0.174	0.366	2.74	3.33E-06	intronic	*PALD1*
2	1,437,163	C	A	0.168	0.376	2.98	6.23E-06	intronic	*TPO*
19	50,189,818	C	G	0.098	0.016	0.15	1.09E-05	intronic	*PRMT1*
20	3,846,843	T	C	0.348	0.172	0.39	1.34E-05	UTR3	*MAVS*
10	72,289,778	T	C	0.109	0.269	3.01	1.82E-05	exonic	*PALD1*
19	50,195,455	A	G	0.092	0.016	0.16	2.38E-05	intronic	*CPT1C*
4	1.85E+ 08	A	G	0.147	0.032	0.19	2.65E-05	intronic	*TRAPPC11*
7	28,449,965	C	T	0.033	0.140	4.82	2.73E-05	intronic	*CREB5*
2	1,442,417	T	C	0.163	0.355	2.82	2.80E-05	intronic	*TPO*
2	1,442,476	C	T	0.163	0.355	2.82	2.80E-05	intronic	*TPO*
5	58,334,645	G	A	0.054	0.199	4.32	3.43E-05	intronic	*PDE4D*
10	72,307,101	C	T	0.603	0.398	2.30	3.58E-05	exonic	*PALD1*
14	35,062,166	T	C	0.114	0.263	2.78	4.50E-05	intronic	*SNX6*
7	1.51E+ 08	G	C	0.315	0.516	2.32	4.78E-05	intronic	*NUB1*
18	14,796,080	A	G	0.234	0.382	2.02	5.39E-05	intronic	*ANKRD30B*
2	1,426,621	A	G	0.332	0.532	2.29	6.31E-05	intronic	*TPO*
9	1.02E+ 08	T	C	0.196	0.065	0.28	6.60E-05	intronic	*GALNT12*
15	23,049,369	A	G	0.337	0.177	0.42	8.36E-05	intronic	*NIPA1*
5	75,427,935	A	G	0.272	0.452	2.21	9.09E-05	exonic	*SV2C*
10	72,288,900	G	A	0.397	0.591	2.20	0.000103	intronic	*PALD1*
10	50,683,438	C	T	0.277	0.118	0.35	0.000104	intronic	*ERCC6*
6	70,970,299	T	C	0.223	0.091	0.35	0.000106	intronic	*COL9A1*

*TPO* encodes a membrane-bound glycoprotein that plays a major role in thyroid gland function. Mutations in this gene are associated with several disorders of thyroid hormonogenesis, i.e., congenital hypothyroidism, and congenital goiter [31, 34]. As depicted in Fig. 2, another SNP within Phosphatase Domain Containing Paladin 1 (*PALD1*) also showed a significant signal. *PALD1* is thought to be involved in the formation of vascular endothelium [36].

### The role of rare variations in SAH risk

It is plausible that analysis of rare variants could explain additional disease risk or trait variability. We next investigated the rare variants across the cohorts by applying the frequency filtering (see Method section). The variants were defined as rare if their frequency in various databases were less than the corresponding threshold (Table S5). Each rare variant was assessed with a specific set of tools to predict their functional impact (see Method section). The 30,651 potential damaging rare variants remained for further analysis.

**Fig. 3:**
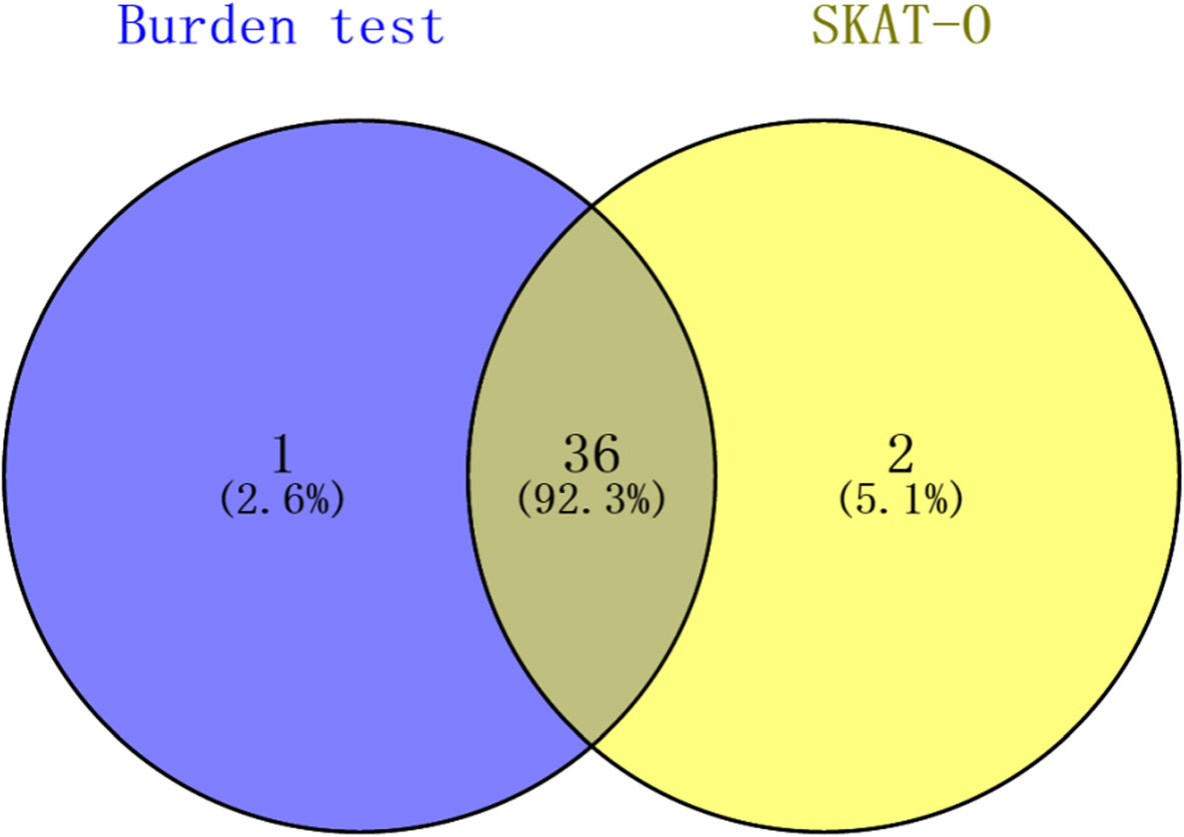
The number of overlapped genes between burden test and SKAT-O analysis

We employed two gene-based methods to identify the susceptibility genes in SAHs. Rare variants burden testing was performed between SAH cases and control samples by Fisher’s exact test, and 38 susceptibility genes, such as gene *OBSCN*, *TJP1*, *ADGRV1*, and *FBN3* etc., were obtained (Table 2). However, when variants with opposite directions of effect in the same gene, the testing power will be reduced. We then employed another analysis with SKAT-O [25] to identify the signals, which both allowed for variants with opposite directions of effect to reside in the same gene. The SKAT-O identified 37 signals (Table 3), which were highly overlap with the results from the burden test (92.3%, Fig. 3), which suggested that these genes could be directly involved in ALS risk.

**Table 2:** Candidate genes in SAH identified by burden test of rare variants

Gene	Number of Cases with mutations	Number of Controls with mutations	Number of Cases without mutations	Number of Controls without mutations	P value	OR
*OBSCN*	26	11	68	82	0.005	2.834
*ABCG8*	7	0	87	93	0.007	Inf
*PIGG*	7	0	87	93	0.007	Inf
*GOLGA2*	6	0	88	93	0.015	Inf
*MTMR4*	6	0	88	93	0.015	Inf
*MYH1*	6	0	88	93	0.015	Inf
*OTOGL*	6	0	88	93	0.015	Inf
*TCF3*	6	0	88	93	0.015	Inf
*TJP1*	10	2	84	91	0.017	5.374
*KMT2C*	8	1	86	92	0.018	8.482
*ADGRV1*	16	6	78	87	0.021	2.958
*FBN3*	9	2	85	91	0.030	4.782
*ABCG5*	5	0	89	93	0.030	Inf
*BCL9*	5	0	89	93	0.030	Inf
*C1orf94*	5	0	89	93	0.030	Inf
*CEBPZ*	5	0	89	93	0.030	Inf
*COG3*	5	0	89	93	0.030	Inf
*CTC1*	5	0	89	93	0.030	Inf
*FDXR*	5	0	89	93	0.030	Inf
*GRIK3*	5	0	89	93	0.030	Inf
*INCENP*	5	0	89	93	0.030	Inf
*IQGAP3*	5	0	89	93	0.030	Inf
*KNDC1*	5	0	89	93	0.030	Inf
*LETMD1*	5	0	89	93	0.030	Inf
*METTL22*	5	0	89	93	0.030	Inf
*NCOA6*	5	0	89	93	0.030	Inf
*PDZD7*	5	0	89	93	0.030	Inf
*PIF1*	5	0	89	93	0.030	Inf
*PLXNA4*	5	0	89	93	0.030	Inf
*RAPGEFL1*	5	0	89	93	0.030	Inf
*RGS14*	5	0	89	93	0.030	Inf
*SCN7A*	5	0	89	93	0.030	Inf
*THEG*	5	0	89	93	0.030	Inf
*VEPH1*	5	0	89	93	0.030	Inf
*ZFP90*	5	0	89	93	0.030	Inf
*LENG8*	7	1	87	92	0.033	7.339
*NIPBL*	7	1	87	92	0.033	7.339
*TECPR2*	7	1	87	92	0.033	7.339

**Table 3:** Candidate genes in SAH identified by SKAT-O analysis

Gene	P value	Number of Marker All	Number of Marker Test	MAC	m	Method bin	MAP
*OBSCN*	0.005	37	36	40	37	ER.A	−1.000
*ABCG8*	0.010	8	7	7	7	ER	0.003
*PIGG*	0.010	9	7	7	7	ER	0.003
*GOLGA2*	0.021	6	6	6	6	ER	0.007
*MTMR4*	0.021	7	7	7	6	ER	0.007
*MYH1*	0.021	6	6	6	6	ER	0.007
*TCF3*	0.021	6	6	6	6	ER	0.007
*KMT2C*	0.026	9	9	9	9	ER	0.001
*FBN3*	0.039	13	12	12	11	ER	0.000
*ABCG5*	0.044	5	5	5	5	ER	0.014
*BCL9*	0.044	5	5	5	5	ER	0.014
*C1orf94*	0.044	5	5	5	5	ER	0.014
*CCDC102A*	0.044	7	5	5	5	ER	0.014
*CEBPZ*	0.044	5	5	5	5	ER	0.014
*COG3*	0.044	4	4	5	5	ER	0.014
*CTC1*	0.044	5	5	5	5	ER	0.014
*FDXR*	0.044	5	5	5	5	ER	0.014
*GRIK3*	0.044	5	5	5	5	ER	0.014
*INCENP*	0.044	3	3	5	5	ER	0.014
*IQGAP3*	0.044	6	5	5	5	ER	0.014
*KNDC1*	0.044	5	5	5	5	ER	0.014
*LETMD1*	0.044	4	4	5	5	ER	0.014
*METTL22*	0.044	4	4	5	5	ER	0.014
*NCOA6*	0.044	5	5	5	5	ER	0.014
*OTOGL*	0.044	5	4	5	5	ER	0.014
*PDZD7*	0.044	3	3	5	5	ER	0.014
*PIF1*	0.044	6	5	5	5	ER	0.014
*PLXNA4*	0.044	4	4	5	5	ER	0.014
*RAPGEFL1*	0.044	3	3	5	5	ER	0.014
*RGS14*	0.044	4	4	5	5	ER	0.014
*SCN7A*	0.044	5	5	5	5	ER	0.014
*THEG*	0.044	5	5	5	5	ER	0.014
*VEPH1*	0.044	5	5	5	5	ER	0.014
*ZFP90*	0.044	4	4	5	5	ER	0.014
*LENG8*	0.050	8	8	8	8	ER	0.002
*NIPBL*	0.050	8	8	8	8	ER	0.002
*TECPR2*	0.050	9	8	8	8	ER	0.002

The overlapped genes were further subjected to functional enrichment analysis. These genes were overrepresented in some pathways related to cellular organization, i.e., adherens junction, and degradation of the extracellular matrix; and transcription factor signal, i.e., TGF-beta signal pathway (Table 4).

**Table 4:** The enriched pathways for the overlapped genes between burden test and SKAT-O analysis

Pathways	Genes
Adherens_junction	*TJP1 PTPRM*
Angiogenesis	*FGFR2 UNC5B NOTCH3 FGFR4 FLT4*
Apelin_signaling_pathway	*NOTCH3 ADCY8*
Cell_cycle_Role_of_SCF_complex_in_cell_cycle_regulation	*FZR1 MAPK8*
Degradation_of_the_extracellular_matrix	*FBN3 ADAMTS18 NCAM1 NTN4*
	*LAMB2 LAMB1 CAPN1 COL20A1 FBLN2*
	*COL14A1 ITGA2 NCAN P4HA2*
Development_TGF-beta_receptor_signaling	*FZR1*
Elastic_fibre_formation	*FBN3 FBLN2*
Endochondral_Ossification	*RUNX2*
ERK_signaling	*MYH1 TCF3 FBN3 ECM2 FGFR2 PRKCQ RPS6KA1 FLT4*
	*TCF19 LAMB2 LAMB1 CAPN1 COL20A1 RASGRP1*
	*NOTCH3 ADCY8 ARHGEF16 CDH12 CDH19 COL14A1 FGFR4*
	*IL12RB1 ITGA2 MAPK8 NCAN NTRK3 PLCD4 ARHGEF2*
HTLV-I_infection	*TCF3 POLE ADCY8 CRTC3 HLA-DPA1 MAPK8*
Integrin_Pathway	*MYH1 FBN3 ECM2 PRKCQ LAMB2 LAMB1 CAPN1*
	*COL20A1 ADCY8 CD36 COL14A1 ITGA2 MAPK8 NCAN*
PAK_Pathway	*MYH1 TCF3 TJP1 FGFR2 NOX4 PRKCQ PTPRH*
	*TCF19 NOTCH3 FGFR4 FLT4 IL12RB1*
	*MAPK8 NTRK3 PLCD4 PTPN3 PTPRM GPLD1*
Sertoli-Sertoli_Cell_Junction_Dynamics	*MYH1 TJP1 SAFB ITGA2 MAPK8 RAB17 RAB34 ARHGEF2*
SMAD_Signaling_Network	*PSMB8 PSMD5*
Smooth_Muscle_Contraction	*NULL*
TGF-beta_receptor_signaling_activates_SMADs	*MTMR4*
TGF-beta_receptor_signaling	*ZFYVE16*
TGF-beta_signaling_pathway_KEGG	*ID4 INHBA SMAD6 ZFYVE16*
TGF-beta_Signaling_Pathways	*MAPK8 RUNX2*

### Pedigree analysis

We performed Mendelian inheritance analysis for two SAH pedigrees with probable inheritance patterns, including (1) dominant inheritance pattern; (2) recessive inheritance pattern; (3) semi-dominant inheritance pattern; (4) compound heterozygote inheritance pattern. Pathogenicity of missense mutations was assumed if predicted pathogenic by at least five out of seven computational methods (SIFT, PolyPhen2, LRT, MutatationTaster, M-CAP, CADD, and REVEL). The potential disease causing variants were only performed in dominant inheritance pattern, and there were 35 and 15 SNPs identified in these two pedigrees, respectively (Table 5 and 6). Twelve and seven candidate genes were identified in pedigree 1 and 2, respectively (Table 7). The gene *COL1A2*, a pathogenic gene in pedigree 2, was also reported to be associated with SAH phenotype [15].

**Table 5:** The potential disease causing SNPs in dominant inheritance pattern for pedigree 1

Chr	Pos	Function	Gene	SIFT	Pp2	LRT	MT	M-CAP	CADD	REVEL
1	2.18E+ 08	exonic	*GPATCH2*	0.00	1.00	D	D	0.23	34.0	0.65
1	2.24E+ 08	exonic	*CCDC185*	0.04	1.00	.	D	0.02	19.6	0.35
1	2.25E+ 08	exonic	*DNAH14*	0.01	0.56	U	D	0.04	23.0	0.09
2	54,609,069	intergenic	*C2orf73 SPTBN1*	0.00	.	.	N	0.00	0.2	0.03
2	55,795,456	exonic	*PPP4R3B*	.	0.39	D	D	0.03	23.6	0.72
2	1.79E+ 08	exonic	*TTN*	0.23	0.02	.	N	0.05	14.1	0.12
2	1.8E+ 08	exonic	*TTN*	0.16	0.80	.	D	0.03	19.0	0.44
2	2.2E+ 08	exonic	*CFAP65*	0.00	0.98	N	D	0.01	34.0	0.19
2	2.2E+ 08	exonic	*STK11IP*	.	.	N	A	.	39.0	.
2	2.23E+ 08	exonic	*PAX3*	0.02	0.22	D	D	0.07	21.4	0.48
3	49,169,107	exonic	*LAMB2*	0.03	0.15	N	D	0.02	22.2	0.11
3	1.83E+ 08	intronic	*ATP11B*	0.03	.	.	N	0.01	5.8	0.02
3	1.94E+ 08	exonic	*ATP13A3*	0.32	0.15	N	N	0.03	6.8	0.25
4	871,443	exonic	*GAK*	0.06	0.42	D	D	0.07	26.3	0.53
4	6,873,370	exonic	*KIAA0232*	0.04	0.20	D	D	0.01	25.1	0.23
4	74,276,089	exonic	*ALB*	0.03	1.00	N	N	0.05	22.4	0.02
7	72,397,374	exonic	*POM121*	0.07	0.51	N	N	0.01	23.2	0.03
7	87,179,859	exonic	*ABCB1*	0.18	0.01	D	D	0.05	14.0	0.26
7	1.17E+ 08	exonic	*CTTNBP2*	0.01	0.99	D	D	0.04	27.4	0.36
7	1.29E+ 08	exonic	*IRF5*	0.21	0.00	N	N	0.03	15.1	0.25
7	1.3E+ 08	exonic	*CPA4*	0.00	0.89	D	D	0.30	25.1	0.31
9	1.31E+ 08	exonic	*ODF2*	0.00	0.99	D	D	0.02	28.3	0.31
12	6,458,130	exonic	*SCNN1A*	0.15	.	.	D	0.10	13.7	0.09
14	95,562,384	exonic	*DICER1*	0.18	0.00	N	N	0.05	0.5	0.02
16	89,865,550	intronic	*FANCA*	0.00	.	.	N	0.01	3.8	.
17	7,231,013	exonic	*NEURL4*	0.00	0.01	D	D	0.04	22.7	0.26
17	7,483,148	exonic	*CD68*	0.00	0.19	N	D	0.02	22.5	0.19
17	7,691,426	exonic	*DNAH2*	0.08	0.43	N	D	0.01	22.5	0.06
17	73,564,902	exonic	*LLGL2*	0.01	0.71	D	D	0.04	27.4	0.53
17	74,085,300	exonic	*EXOC7*	0.22	0.02	D	D	0.00	17.8	0.08
20	55,777,539	exonic	*BMP7*	0.01	0.74	D	D	0.08	29.0	0.22
21	46,929,308	exonic	*COL18A1*	0.14	0.16	N	N	0.10	10.5	0.12
22	30,074,259	exonic	*NF2*	0.59	0.02	D	D	0.08	18.4	0.43
22	50,721,594	exonic	*PLXNB2*	0.51	0.00	N	N	0.05	14.3	0.27
22	50,945,311	exonic	*LMF2*	0.00	0.99	D	D	0.67	28.0	0.41

**Table 6:** The potential disease causing SNPs in dominant inheritance pattern for pedigree 2

Chr	Pos	Function	Gene	SIFT	Pp2	LRT	MT	M-CAP	CADD	REVEL
1	1.5E+ 08	exonic	*HIST2H2AC*	0.00	0.10	N	D	0.024	8.0	0.15
1	1.52E+ 08	exonic	*RPTN*	0.01	0.02	.	N	0.003	16.3	0.01
1	1.57E+ 08	exonic	*IQGAP3*	0.00	1.00	D	D	0.154	35.0	0.84
1	1.62E+ 08	exonic	*DUSP12*	0.10	1.00	D	D	0.009	23.4	0.16
6	56,471,328	intronic	*DST*	0.01	0.17	N	.	0.033	12.0	0.10
7	20,782,555	exonic	*ABCB5*	0.00	0.99	D	D	0.039	27.8	0.70
7	29,132,261	exonic	*CPVL*	0.04	0.99	N	D	0.142	26.6	0.47
7	94,057,039	exonic	*COL1A2*	0.10	0.98	D	N	0.082	26.2	0.44
7	1.01E+ 08	exonic	*MUC17*	0.02	0.61	.	N	0.003	5.6	0.04
7	1.51E+ 08	exonic	*CHPF2*	0.03	0.89	D	D	0.049	23.3	0.32
8	90,936,937	exonic	*OSGIN2*	0.38	0.03	D	D	0.01	11.3	0.07
17	3,030,476	exonic	*OR1G1*	0.05	0.01	.	N	0	13.6	0.03
17	4,619,845	exonic	*ARRB2*	0.04	0.90	D	D	0.034	32.0	0.15
17	6,683,525	exonic	*FBXO39*	0.14	0.09	D	D	0.024	19.8	0.17
17	7,733,695	exonic	*DNAH2*	0.01	0.89	N	N	0.004	23.8	0.11

**Table 7:** The candidate genes in two pedigrees

Candidate genes in pedigree 1	Candidate genes in pedigree 2
*GPATCH2*	*IQGAP3*
*CFAP65*	*DUSP12*
*PAX3*	*ABCB5*
*GAK*	*CPVL*
*KIAA0232*	*COL1A2*
*CTTNBP2*	*CHPF2*
*CPA4*	*ARRB2*
*ODF2*	
*NEURL4*	
*LLGL2*	
*BMP7*	
*LMF2*	

### Putative pathogenic genes for SAHs

Protein–protein interactions were known as mediating many cellular functions, including cell cycle progression, signal transduction, and metabolic pathways. The genes that interacted with the known SAH genes may influence the SAH phenotypes by participating in the same network/pathway. Basic on previous studies, we collected 28 SAH associated genes (Table S6), and these genes were further assessed the direct and indirect associations with other genes by STRING [35]. In total, we identified 47 putative interacted genes with the SAH (Table S7). To look deep into the pathogenic genes associated with SAH, we selected the overlapped genes among the results from the burden test, SKAT-O analysis and putative interacted genes (Table 8). Finally, we identified 25 putative pathogenic genes for SAH.

**Table 8:** The overlapped genes among the results from burden test, SKAT-O analysis and PPI analysis

Candidate pathogenic genes	
*MYH1*	*CD36*
*TJP1*	*COL14A1*
*FGFR2*	*FGFR4*
*NCAM1*	*FLT4*
*NOX4*	*FZR1*
*RPS6KA1*	*ID4*
*LAMB2*	*IL12RB1*
*LAMB1*	*ITGA2*
*COL20A1*	*MAPK8*
*FBLN2*	*NCAN*
*POLE*	*P4HA2*
*NOTCH3*	*RUNX2*
*ADCY8*	

Among these genes, *FBLN2* has been identified a member of fibulin family, and is responsible for maintenance of the adult vessel wall after injury [8]. *BMP7* was reported to play an important role in facilitating recovery after stroke in rat [9]. *ITGA2* is responsible for adhesion of platelets and other cells to collagens and organizations of extracellular matrix. Previous study demonstrated *ITGA2*-deficient mice overexpressed transforming the growth factor TGFβ [16, 19], which was known to be highly associated with aortic aneurysm and IA. Moreover, both *ITGA2* and *TTN* were involved in hemostasis [3, 30]. Notably, Notch signaling plays a pivotal role during vascular development [4, 18]. Mutations in *NOTCH3* have been identified as the underlying cause of cerebral autosomal dominant arteriopathy with subcortical infarcts and leukoencephalopathy (CADASIL), the most common inherited stroke and dementia syndrome in the group of degenerative small vessel diseases [23]. Our findings demonstrated the therapeutic potential of modifying these signaling in SAHs.

## Discussion

Subarachnoid hemorrhage (SAH) is the rarest but most fatal type of stroke, identification of genetic variants that confer susceptibility to SAH is clinically important to prevent it [20]. In the present study, we performed WES for SAH cases and controls, to identify causal variations that associated with SAH risk in China, which enabled us to systemically evaluate protein-altering variants and candidate functional genes.

Across GWAS with a total of 188 samples, we found a genome-wide significant association of SNPs in *TPO* and *PALD1* with SAH risk. These two genes are involved in disorders of thyroid hormonogenesis and formation of vascular endothelium, respectively. Previous studies of IA have identified *SERPINA3* (rs4934) as associated risk loci in the Finnish population, and *CSPG2* (rs251124) and *HSPG2* (rs3767137) loci as susceptibility sites in the Dutch population. However, in our cohorts, there was no significantly associated signal in these genes, which may due to the different genetic background among the populations.

We then investigated the role of low-frequency variants of intermediate effect in SAH risk through rare SNPs analysis. The pathogenic genes with rare, damaging SNPs were enriched in some pathways related to cellular organization, i.e., degradation of the extracellular matrix; and transcription factor signal, i.e., TGF-beta signaling pathway. TGF-beta signaling plays a vital role in vasculogenesis and maintenance of blood vessel, and is involved in aortic aneurysm and IA. These results highlight the functional importance of rare variations in SAH risk.

The two pedigree samples were mainly used to performed Mendelian inheritance analysis, and it revealed autosomal dominant inheritance of pathogenic genes. In the same time, some potential disease causing variants were also found, such as the gene *COL1A2* which was reported to be associated with SAH phenotype [15]. Combing the results from the network analysis of known SAH-associated genes, we obtained a list of candidate susceptibility genes. Among these genes, several were demonstrated to be associated with maintenance of blood vessel, including *FBLN2*, *ITGA2*, *BMP7*, and *NOTCH3*. *NOTCH3* is known to be associated with the most common inherited stroke, CADASIL. These potential targets needed to be further validated in experiment models both in vivo and in vitro, which may facilitate to develop clinical strategies for early detection and intervention.

In conclusion, we have identified a key role for rare variations in SAH and discovered SNPs in new complex loci. However, there are still some limitations to our current study due to the small sample size and availability of family genetic data. In future, the identified candidate genes, i.e., *TPO*, *PALD1* and *ITGA2,* will be necessary to validate in independent study populations or a larger sample size for Chinese population. Determination of genotypes for SNPs in these genes will guide the development of therapeutic strategies for SAH.

## Supplementary information

**Additional file 1: Table S1.** Sample background information. **Table S2.** Basic information of sequencing. **Table S3.** reads mapping statistics. **Table S4.** Variation distribution statistics. **Table S5.** Variation filtering threshold. **Table S6.** Known genes in SAH. **Table S7.** Interacted genes with known genes in SAH

## Data Availability

All data and materials mentioned in this article are available. The original data have been submitted to the public data depository CNGB Nucleotide Sequence Archive (CNSA;https://db.cngb.org/cnsa/) of the China National GeneBank DataBase (CNGBdb) with accession number CNP0000954.
